# Therapeutic respiratory and functional rehabilitation protocol for intensive care unit patients affected by COVID-19: a structured summary of a study protocol for a randomised controlled trial

**DOI:** 10.1186/s13063-021-05210-y

**Published:** 2021-04-12

**Authors:** Ana Cristina Carvalho, Jorge Moreira, Pedro Cubelo, Pedro Cantista, Catarina Aguiar Branco, Bruno Guimarães

**Affiliations:** 1 Department of Public Health - USP Porto Oriental, ACES Grande Porto VI, Porto, Portugal; 2 Department of Physical and Rehabilitation Medicine - Centro Hospitalar de Entre o Douro e Vouga, Santa Maria da Feira, Portugal; 3 Department of Physical and Rehabilitation Medicine - Centro Hospitalar Universitário do Porto, Porto, Portugal; 4 Department of PRM/ Integrated Clinic, Faculty of Dental Medicine, University of Porto, Porto, Portugal; 5 Department of Public Health, Forensic Sciences and Medical Education, Faculty of Medicine, University of Porto, Porto, Portugal; 6 Department of Surgery and Physiology, Faculty of Medicine, University of Porto, Porto, Portugal; 7 Cardiovascular Research Center, Faculty of Medicine, University of Porto, Porto, Portugal

**Keywords:** COVID-19, Randomized controlled trial, protocol, intensive care unit, post-intensive care syndrome, functional rehabilitation, respiratory rehabilitation

## Abstract

**Objectives:**

The primary objective of the presented study is to analyze the respiratory and functional effects of a rehabilitation program in patients affected by hospitalization in Intensive Care Unit (ICU) due to COVID-19, in comparison with the group treated with standard of care, at discharge endpoint.

The secondary objectives of the presented study are to evaluate different outcomes of the rehabilitation program in comparison to standard of care regarding: functional performance at 4-week and 12-week post- discharge mark; health-related quality of life, the impact on the health services (namely days of hospitalization), the cost-effectiveness of the intervention proposed.

**Trial design:**

This is a randomized, controlled, double-blind, double-arm clinical trial of treatment, with an allocation ratio 1:1 and framework of superiority.

**Participants:**

The study will be conducted at Centro Hospitalar Entre Douro e Vouga, Santa Maria da Feira, Portugal. Potential participants will be adult patients (≥18 years old) hospitalized in ICU with respiratory insufficiency due to COVID-19, who are referred to respiratory and functional rehabilitation. Only patients approved by physical rehabilitation doctors to perform respiratory and functional rehabilitation will be considered potential participants. To be eligible for inclusion participants must have been independent in their activities of daily living before the onset of critical illness (verbal statement by their proxy) and have to meet the safety criteria defined by the Portuguese Society of Physical Rehabilitation Medicine.

**Intervention and comparator:**

Both groups will receive usual medical and nursing care in the ICU, which involves assessment and treatment of the respiratory system and may include positioning, hyperinflation techniques and suctioning. The physical function of the patient is assessed, and active bed exercises and mobility are encouraged as soon as possible and may include sitting out of bed.

The intervention group will receive a functional and respiratory multidisciplinary rehabilitation protocol (that includes medical, nursing, physiotherapy and occupational therapy interventions) during their entire hospital stay.

After reassurance that the patients fulfil the safety criteria, they will initiate the rehabilitation protocol, individualized to each patient based on the clinical status. The rehabilitation interventions and exercises implemented will be consistent with recommendations from the Portuguese Society of Physical Rehabilitation Medicine.

The intervention will occur 6 days per week (Monday to Saturday), fifteen minutes, twice per day for each participant. Throughout all activities, progression will be increased successively, depending on the individual’s tolerance and stability.

After discharge, the intervention group will continue with rehabilitation exercises, prescribed by physical rehabilitation doctors. These exercises are designed for the patient to do at home, and then report their execution to rehabilitation nurses through teleconsultation, until 12 weeks after ICU discharge.

**Main outcomes:**

Baseline descriptive data collection will include age, sex, comorbidities and date of admission to ICU. The need of mechanical ventilation and length of use, as well as the need for oxygen therapy, length of ICU stay (days/hours), incidence of ICU readmission, discharge destination and survival will also be recorded.

Prior to intervention, every two days and at discharge, participants will be evaluated using the following scales: Glasgow Coma Scale, Richmond Agitation Sedation Scale, Chelsea Critical Care Physical Assessment, 5 standardized questions for cooperation, Medical Research Council Sum-Score, Handgrip strength test and Medical Research Council dyspnea scale. At discharge, Borg Rating of Perceived Exertion will be evaluated.

The primary outcome measure will be functional capacity using the 6-Minute Walk Test, and it will be measured at discharge and at the 4-week and 12-week mark. Medical Research Council Sum-Score, Handgrip strength test, Medical Research Council dyspnea scale and Borg Rating of Perceived Exertion will also be re-evaluated at the 4-week and 12-week mark. The health related quality of life will also be used as an outcome measure, using the 12-Item Short Form Survey, at 12 weeks of follow-up.

**Randomisation:**

Participants will be divided into two groups, standard care and intervention, by means of balanced randomization at a 1:1 ratio using blocks of 10 participants. The randomization sequence is going to be created using a free software (http://www.randomized.org/). In order to ensure the confidentiality of the randomisation sequence, this process will be conducted by an assessor external to the study.

**Blinding (masking):**

The evaluators in the study will be blinded during the entire process. The evaluators will be unaware of the study objectives and the randomized distribution of patients to study groups and will not have access to the randomization sequence. Although blinding for patients will not be possible to achieve completely, subjects will be unaware of other treatment modalities, and they will not know if they belong to the intervention or standard group. As for the treating physiotherapists and ICU staff, blinding will not be possible to achieve, but they will not be responsible for assessing outcomes.

**Numbers to be randomised (sample size):**

We plan to randomise 40 participants to each group. 80 participants in total.

**Trial Status:**

This is the second and definitive protocol version, dated from 26th February 2021. Recruitment started on 8^th^ March 2021. Participants will be recruited between March 8, 2021, and June 8, 2021. Study completion is expected to be October 2021.

**Trial registration:**

ReBEC RBR-7rvhpq9. Registry name: The effect of rehabilitation in hospitalized COVID-19 patients. Registered on 17 March 2021.Retrospectively registered.

**Full protocol:**

“The full protocol is attached as an additional file, accessible from the Trials website (Additional file 1). In the interest in expediting dissemination of this material, the familiar formatting has been eliminated; this Letter serves as a summary of the key elements of the full protocol”.

**Supplementary Information:**

The online version contains supplementary material available at 10.1186/s13063-021-05210-y.

## Supplementary Information

**Additional file 1.** Full study protocol.

## Data Availability

The datasets analyzed during the current study are available from the corresponding author on reasonable request. The data will be available after the main publication of them; for other circumstances, they should consult the corresponding author. Any data required to support the protocol can be supplied on request.

