# Supplementary material for: Therapeutic respiratory and functional rehabilitation protocol for intensive care unit patients affected by COVID-19: a structured summary of a study protocol for a randomised controlled trial
Source: Trials. 2021 Apr 12;22:268. doi: 10.1186/s13063-021-05210-y (PMC8039799; doi:10.1186/s13063-021-05210-y)
Supplement: Supplementary file 1 — Additional file 1. Full study protocol. [file 13063_2021_5210_MOESM1_ESM.docx]

**Therapeutic respiratory and functional rehabilitation protocol for intensive care patients affected by COVID-19: study protocol for a randomized controlled trial**

**Ana Cristina Carvalho^1*^, Jorge Moreira^2^, Pedro Cubelo^2^, Pedro Cantista^3^, Catarina Aguiar Branco^2,4^, Bruno Guimarães^2,5,6,7*^**

^1^ Public Health Unit - Porto Oriental, ACeS Grande Porto VI. Porto, Portugal.

^2^ Department of Physical and Rehabilitation Medicine - Centro Hospitalar de Entre o Douro e Vouga. Santa Maria da Feira, Portugal.

^3^ Department of Physical and Rehabilitation Medicine - Centro Hospitalar Universitário do Porto. Porto, Portugal.

^4^ Department of PRM/ Integrated Clinic, Faculty of Dental Medicine, University of Porto. Porto, Portugal.

^5^ Department of Public Health, Forensic Sciences and Medical Education, Faculty of Medicine, University of Porto. Porto, Portugal.

^6^ Department of Surgery and Physiology, Faculty of Medicine, University of Porto. Porto, Portugal.

^7^ Cardiovascular Research Center. Faculty of Medicine, University of Porto. Porto, Portugal.

**Abstract**

**Background:** The coronavirus disease 2019 (COVID-19), declared pandemic on 11^th^ March 2020 by the World Health Organization, has led to an increasing number of patients in the intensive care units (ICU) around the world. The size of this post-ICU cohort will be unprecedented, with many patients vulnerable to post-intensive care syndrome. The implementation of rehabilitation programs that begin in the ICU may play a role in reducing these harmful consequences. The primary objective of the presented study is to analyze the respiratory and functional effects of a rehabilitation program in patients affected by the COVID-19 hospitalized in ICU, in comparison with the group subjected to standard of care, at discharge endpoint.

**Methods:** A randomized, controlled, double-blind, double-arm clinical trial will be conducted in Centro Hospitalar Entre Douro e Vouga. Patients who fulfil the eligible criteria will be recruited and randomized to one of two groups: standard of care and intervention group. The intervention group will receive a functional and respiratory rehabilitation protocol. Baseline demographic and clinical data will be collected. Functional and respiratory capacities will be evaluated using the following scales: Glasgow Coma Scale, Richmond Agitation Sedation Scale, Chelsea Critical Care Physical Assessment, 5 standardized questions for cooperation, Medical Research Council sumscore, Handgrip strength test, Medical Research Council dyspnea scale and Borg Rating of Perceived Exertion. The primary outcome measure evaluated at discharge, 4-week and 12-week of follow-up, is functional capacity using the 6-Minute Walk Test. The health related quality of life will be evaluated at 12-week mark after discharge, using the 12-Item Short Form Survey.

**Discussion:** In this prospective, single-center, allocation-concealed and assessor-blinded randomized controlled trial we expect the intervention group to achieve better functional and respiratory outcomes, less days of hospitalization and an improvement in health related quality of life, comparing to the standard of care group.

**Trial registration:** RBR-7rvhpq9. Retrospectively registered.

**Keywords:** COVID-19, Randomized controlled trial, protocol, intensive care unit, post-intensive care syndrome, functional rehabilitation, respiratory rehabilitation

**Introduction**

In December 2019, a group of pneumonia cases was reported in Wuhan, China, which was later found to be caused by a novel coronavirus, temporarily named 2019-nCoV (1-3). Later, it was found that the new coronavirus belongs to a “species” category called severe acute respiratory syndrome-related coronavirus (SARS-CoV) and is related to the virus that swept China in 2003 (3). Therefore, the virus was renamed as the severe acute respiratory syndrome coronavirus 2 (SARS-CoV-2), and the World Health Organization (WHO) named the disease caused by the SARS-CoV-2 as coronavirus disease 2019 (COVID-19) (4). Since then, the infection has spread all over the world, and on the 11th March 2020, WHO declared the state of pandemics, which led to unprecedented measures all over the world, namely social isolation, prevention of contacts and changes in the healthcare organization (5). Prior to the implementation of any public health interventions, the basic reproduction number of SARS-CoV-2 was estimated to be as high as 5,7, meaning that each infected individual had the potential to transmit the disease to as many as 5 to 6 new individuals (6). As of 27^th^ January 2021, the virus has resulted in over 98,2 million cases and over 2,1 million deaths all over the world, with significantly worse outcomes and higher mortality in the elderly (7).

The most common symptoms of COVID-19 are fatigue, fever, dry cough, and anosmia (2, 4, 8-10). According to the WHO (4), COVID-19 can manifest as mild, moderate, severe, and critical disease. Approximately 80% of cases are mild and self-limited, primarily affecting the upper airway with limited involvement of the lungs (9-11). Moderate disease is characterized by the evidence of pneumonia (9-11). Severe infection, characterized by dyspnea, tachypnea, hypoxemia, cardiovascular sequalae, and extensive lung disease, occurs in 15% of cases (9-11). Critical infection, characterized by respiratory failure, septic shock, and multisystem organ dysfunction, and often complicated by acute respiratory distress syndrome (ARDS) and disseminated intravascular coagulopathy (DIC), occurs in 5% of cases (9-11). This small percentage of COVID-19’ patients requires intensive care and reliance on ventilators, but, unlike other diseases, when these patients need this kind of treatment, they will need it for a longer period than more typical uses of intensive care unit (ICU) (12). This raises a major concern: the recovery from the consequences of severe respiratory illness and the secondary disabilities that result from intensive care treatments (12).

In 2012, the Society of Critical Care Medicine held a stakeholder conference to address subacute/chronic physical and psychological problems after ICU discharge, in which post-intensive care syndrome (PICS) was proposed (13). PICS refers to the disability that persists in the patients who survives critical illness, and it is a physical, cognitive, and mental disorder that occurs during ICU stay or after ICU or hospital discharge and includes the long term prognosis of ICU patients and effects on the patient's family (13-15). PICS is recognized as a growing public health burden due to the associated neuropsychological and functional disability (16). The cognitive impairment and psychiatric disorders clearly have substantial direct and indirect economic effects, not only for the patients themselves, but also for their families (17). These impairments have a negative impact on work productivity, work capacity and life satisfaction, as well as they increase health-related medical costs (16).

Patients with PICS may have a variety of symptoms, including functional, respiratory and cognitive symptoms (14, 15). These symptoms start after the critical illness, persist after discharge from the ICU and can last for weeks, months and even years. Intensive care unit-acquired weakness (ICU-AW) is one factor related to muscle weakness (18, 19). It is defined as the acute muscle weakness of the extremities in a symmetric pattern, which is caused by critical illness, and it has an important contribution to prolonged mechanical ventilation, increased ICU and hospital lengths of stay, and mortality (18, 19). Currently, there is no consensual intervention applied to improve the outcomes of ICU-AW, but early physical rehabilitation, neuromuscular electrical stimulation and glucose control are some of the preventive measures that have been applied (20). PICS components should be identified before ICU discharge and rehabilitation should be started as soon as possible (17).

The level of health burden associated with PICS suggests that the prevention of PICS symptoms from ICU admission is more important and effective than intensive treatment of PICS following ICU discharge (14). There is no consensus in the literature about the beneficial effects of rehabilitation on PICS, with some studies showing beneficial effects, and others showing no effects (20-22). Also, the dose–response of physical rehabilitation for clinical outcomes is unknown (14). Therefore, more randomized controlled trials are needed to clarify the effect of physical rehabilitation on PICS.

As SARS-CoV-2 is spreading through the world, the number of patients that have been subjected to ICU treatment and survive is increasing due to the success of critical care medicine in reducing mortality (23). The size of this post-ICU cohort will be unprecedented, with a large number of patients vulnerable to PICS and other consequences of intensive care (12, 23). Therefore, it is of great importance that the medical community apply all the resources available to optimize the COVID-19 survivorship experience and lessen the impact of PICS on the health burden, and the implementation of rehabilitation programs that begin in the ICU appears to be one of the most promising tool for this purpose (23, 24).

**Objectives**

The primary objective of the presented study is to analyze the respiratory and functional effects of a rehabilitation program in patients affected by the COVID-19 hospitalized in ICU, in comparison with the group subjected to standard of care, at discharge endpoint.

The secondary objectives of the presented study are to evaluate different outcomes of the rehabilitation program in comparison to standard of care regarding:

- Functional performance at 4-week and 12-week post- discharge mark,
- Health-related quality of life.
- The impact on the health services (namely days of hospitalization),
- The cost-effectiveness of the intervention proposed.

**Methods and Design**

We will conduct a controlled, randomized, double-blind, double-arm clinical trial of treatment in the tertiary, interdisciplinary ICU of Centro Hospitalar Entre Douro e Vouga. The study has been approved by the Commission of Ethics for Health of Centro Hospitalar Entre Douro e Vouga, reference CA-081/2021-0t_MP/CC. This trial adheres to the recommendations from the Consolidated Standards of Reporting Trials (CONSORT) statement (25).

**Participants**

Potential participants will be adult patients (≥18 years old) hospitalized at ICU of Centro Hospitalar Entre Douro e Vouga, with respiratory insufficiency due to COVID-19, who are referred to respiratory and functional rehabilitation. Only patients approved by physical rehabilitation doctors to perform respiratory and functional rehabilitation will be considered potential participants. To be eligible for inclusion, participants must have been independent in their activities of daily living before the onset of critical illness (verbal statement by their proxy) and have to meet the safety criteria defined by the Portuguese Society of Physical Rehabilitation Medicine (26) namely:

1. Assessment of respiratory system
   1. Respiratory Rate equal to or less than 30 breaths/min
   2. Oxygen Saturation equal to or greater than 90%
   3. Inspired oxygen concentration less than or equal to 60%
   4. Positive end-expiratory pressure less than or equal to 10 cmH_2_O
   5. Absence of dyssynchrony between ventilator and patient
2. Assessment of cardiovascular system
   1. Systolic Blood Pressure between 90 and 180 mmHg
   2. Mean Arterial Pressure between 65 mmHg and 110 mmHg
   3. Blood Pressure variability less than 20%
   4. Heart rate between 40 and 120 beats per minute
   5. Absence of arrhythmias or rhythm abnormalities *de novo* with impact on hemodynamic stability
   6. No new antiarrhythmic drugs in the last 24 hours
   7. No signs of recent acute coronary syndrome, particularly in the last 48 hours
   8. No continuous perfusion of vasopressors or no recent increase in the dose of vasopressors, namely in the last 2 hours
   9. No continuous perfusion of vasodilators
   10. No signs of shock accompanied by serum lactate greater than or equal to 4 mmol/L
   11. Absence of deep venous thrombosis or pulmonary thromboembolism in the last 24 hours and, in the presence of these entities, with anticoagulation instituted at least 24 hours ago
   12. Absence of severe aortic stenosis with impact on hemodynamic stability
3. Assessment of neurological system
   1. Intracranial pressure less than 20 cmH_2_O
   2. No need for increased sedation in the last 30 minutes
4. Hematological and laboratory evaluation
   1. Stable hemoglobin level greater than 7 g/dL
   2. Platelet level above 20000 / mm^3^
5. Other safety criteria:
   1. No need of prone position
   2. Absence of unstable limb or spine fracture
   3. No signs of hepatic or kidney failure
   4. No signs of active bleeding
   5. Body temperature between 36 and 38.5 ° C

Potential participants will be excluded if they don’t met the safety criteria. Further exclusion criteria are prior muscle weakness (such as a preexisting neurological or neuromuscular disease), prior pulmonary diseases that condition forced expiratory volume on 1 second (FEV1) and/or Tiffeneau-Pinelli index (such as pulmonary obstructive disease or restrictive pulmonary diseases), acute thrombosis, contraindications to cycling (such as fractures or recent surgical procedures to the lower limbs, preexisting open wounds, and body weight of more than 120 kg) and patients with a diagnosis on admission that excludes the possibility of walking at hospital discharge.

**Interventions**

Both groups will receive usual medical and nursing care in the ICU, which involves assessment and treatment of the respiratory system and may include positioning, hyperinflation techniques and suctioning. The physical function of the patient is assessed, and active bed exercises and mobility are encouraged as soon as possible and may include sitting out of bed.

The intervention group will receive a functional and respiratory multidisciplinary rehabilitation protocol (that includes medical, nursing, physiotherapy and occupational therapy interventions) during their entire hospital stay.

After reassurance that the patients fulfil the safety criteria, they will initiate the rehabilitation protocol, individualized to each patient based on the clinical status. The rehabilitation interventions and exercises implemented will be consistent with recommendations from the Portuguese Society of Physical Rehabilitation Medicine (26).

The intervention will occur 6 days per week (Monday to Saturday), fifteen minutes, twice per day for each participant. Throughout all activities, progression will be increased successively, depending on the individual’s tolerance and stability.

All adverse events will be noted, which includes any event that occurs during or up to fifteen minutes, after intervention, and persists despite therapy interruption and constitute criteria for discontinuing intervention.

After discharge, the intervention group will continue with rehabilitation exercises, prescribed by physical rehabilitation doctors. These exercises are designed for the patient to do at home, and then report their execution to rehabilitation nurses through teleconsultation, until 12 weeks after ICU discharge.

**Outcomes**

Baseline descriptive data collection will include age, sex, comorbidities and date of admission to ICU. The need of mechanical ventilation and length of use, as well as the need for oxygen therapy, length of ICU stay (days/hours), incidence of ICU readmission, discharge destination and survival will also be recorded.

Prior to intervention, participants will be evaluated using the following scales:

- Glasgow Coma Scale (GCS) – it is based on motor responsiveness, verbal performance, and eye opening to appropriate stimuli, and was designed and should be used to assess the depth and duration coma and impaired consciousness (27).
- Richmond Agitation Sedation Scale (RASS) - it is a commonly utilized and validated sedation assessment scale, that was designed to assess the level of alertness and agitated behavior in critically ill patients (28). It is a 10-point scale ranging from -5 to +4 (28). Levels -1 to -5 denote 5 levels of sedation, starting with “awakens to voice” and ending with “unarousable” (28) Levels +1 to +4 describe increasing levels of agitation (28). The lowest level of agitation starts with apprehension and anxiety, and peaks at combative and violent. RASS level 0 is “alert and calm” (28).
- Chelsea Critical Care Physical Assessment (CPAx) – it is a test used on patients in ICU to assess physical and respiratory function impairments and morbidity (29). It is a pictorial composite of 10 numerical evaluations of pertinent functions and impairments, namely, respiratory function, cough, moving within the bed, supine to sitting on the edge of the bed, dynamic sitting, standing balance, sit to stand, transferring from bed to chair, stepping, grip strength (29).
- 5 standardized questions for cooperation (S5Q) – it assesses patients’ ability to cooperate, and it consists of five commands, and 1 point is attributed for each command that the patient is able to perform (30).
- Medical Research Council sum score (MRC-SS) - it evaluates global muscle strength. Manual strength of six muscle groups (shoulder abduction, elbow flexion, wrist extension, hip flexion, knee extension, and ankle dorsiflexion) is evaluated on both sides using MRC scale (31). Summation of scores gives MRC-sum score, ranging from 0 to 60 (31).
- Handgrip strength test – it uses a handgrip dynamometer to assess muscle strength (31).
- Medical Research Council dyspnea scale (mMRC) – it is the test mot frequently used in clinical practice to evaluate dyspnea (32). It summarizes the score of five offered statements about breath possibility during the daily activities, and patients choose the one which describes their problems in the best manner (32). It is easy to perform and correlates with clinical parameters and parameters of respiratory function (32).

Every two days and at discharge of intervention the following scales will be re-evaluated: GCS, RASS, CPAx, S5Q, MRC-SS, handgrip strength test, mMRC. At discharge, Borg Rating of Perceived Exertion (BRPE) will be evaluated. It is a widely used and reliable indicator to monitor and guide exercise intensity, that allows individuals to subjectively rate their level of exertion during exercise or exercise testing (33). MRC-SS, BRPE, handgrip strength test and mMRC will also be evaluated at 4-week and 12-week mark.

The primary outcome measure evaluated at discharge, as well as, in the follow-up at 4-week and 12-week mark, is functional capacity using the 6-Minute Walk Test (6MWT). The 6MWT is a practical and simple test that measures the distance a person can quickly walk on a flat, hard surface in 6 minutes (34). It is self-paced and submaximal and reflects the functional exercise level for daily physical activities (34).

The health related quality of life (HRQoL) will be evaluated at 12-week mark after discharge using 12-Item Short Form Survey (SF-12) (35, 36). It is a self-reported outcome measure assessing the impact of health on an individual's everyday life (35). It has eight domains: limitations in physical activities because of health problems, limitations in social activities because of physical or emotional problems, limitations in usual role activities because of physical health problems, bodily pain, general mental health (psychological distress and well-being), limitations in usual role activities because of emotional problems, vitality (energy and fatigue) and general health perceptions (35).

The data will be collected by evaluators attached to the research group who have previously been instructed in the procedures to follow and do not know the group to which the patients belong.

The schedule for enrolment, data collection, intervention and outcome measures is described on table 1.

**Data for the economic analysis**

A cost-consequences economic evaluation will evaluate the effectiveness of the intervention compared to standard of care practice. Costs will be incurred at the patient level, in delivering the intervention. The cost of intervention refers to the resources applied to the implementation of the rehabilitation program to individual patient care. Variable resource use associated with the intervention (time spent, number of resources) will be recorded for each patient.

The economic impact related to the hospitalization of the analyzed patients was calculated based on the Case Mix Groups (CMG™), according to the Portuguese health system guidance. The CMG system defines and identifies homogeneous groups of inpatient based on most responsible diagnosis and taking in consideration the age, length of stay, and health care resources.

**Sample size**

The sample size was estimated using the Winpepi® program (http:// [www.brixtonhealth.com/pepi4windows.html](http://www.brixtonhealth.com/pepi4windows.html)), with an estimated alfa risk of 5%, a power of 95%, a minimum expected difference of 10%, a rate of follow-up losses of 10%, a proportion in group B (standard of care) of 20% and proportion in group A (intervention) of 60%, for which 40 subjects are required in each group. We will include 80 patients who will be divided into two groups.

**Recruitment, Randomization and Blinding**

The participants will be recruited using the hospital computer system, after consultation of patient identification, confirmation of medical diagnosis (respiratory failure with a confirmed PCR test for SARS-CoV-2 positive), and current clinical condition by an assessor, who is going to evaluate patient’s eligibility.

Reasons for nonrecruitment will be noted in a daily screening log. Eligible patients will be asked to participate. Written informed consent from the patient or their next of kin will be sought. If the next of kin provides initial consent, the participant will be asked to consent once they are able to do so. Trial participation will have no effect on routine management of their underlying condition or any other healthcare requirements. Participants have the right to withdraw from the trial at any given time. If unwilling to participate or withdrawing from the trial, patients will be excluded from the study and asked if the data that has already been collected may be retained.

Participants will be then divided into two groups, standard care and intervention, by means of balanced randomization at a 1:1 ratio using blocks of 10 participants. The randomization sequence is going to be created using a free software (<http://www.randomized.org/>). In order to ensure the confidentiality of the randomization sequence, this process will be conducted by an assessor external to the study.

The evaluators in the study will be blinded during the entire process. The evaluators will be unaware of the study objectives and the randomized distribution of patients to study groups and will not have access to the randomization sequence. Although blinding for patients will not be possible to achieve completely, subjects will be unaware of other treatment modalities, and they will not know if they belong to the intervention or standard group. As for the treating physiotherapists and ICU staff, blinding will not be possible to achieve, but they will not be responsible for assessing outcomes.

**Participants’ timeline**


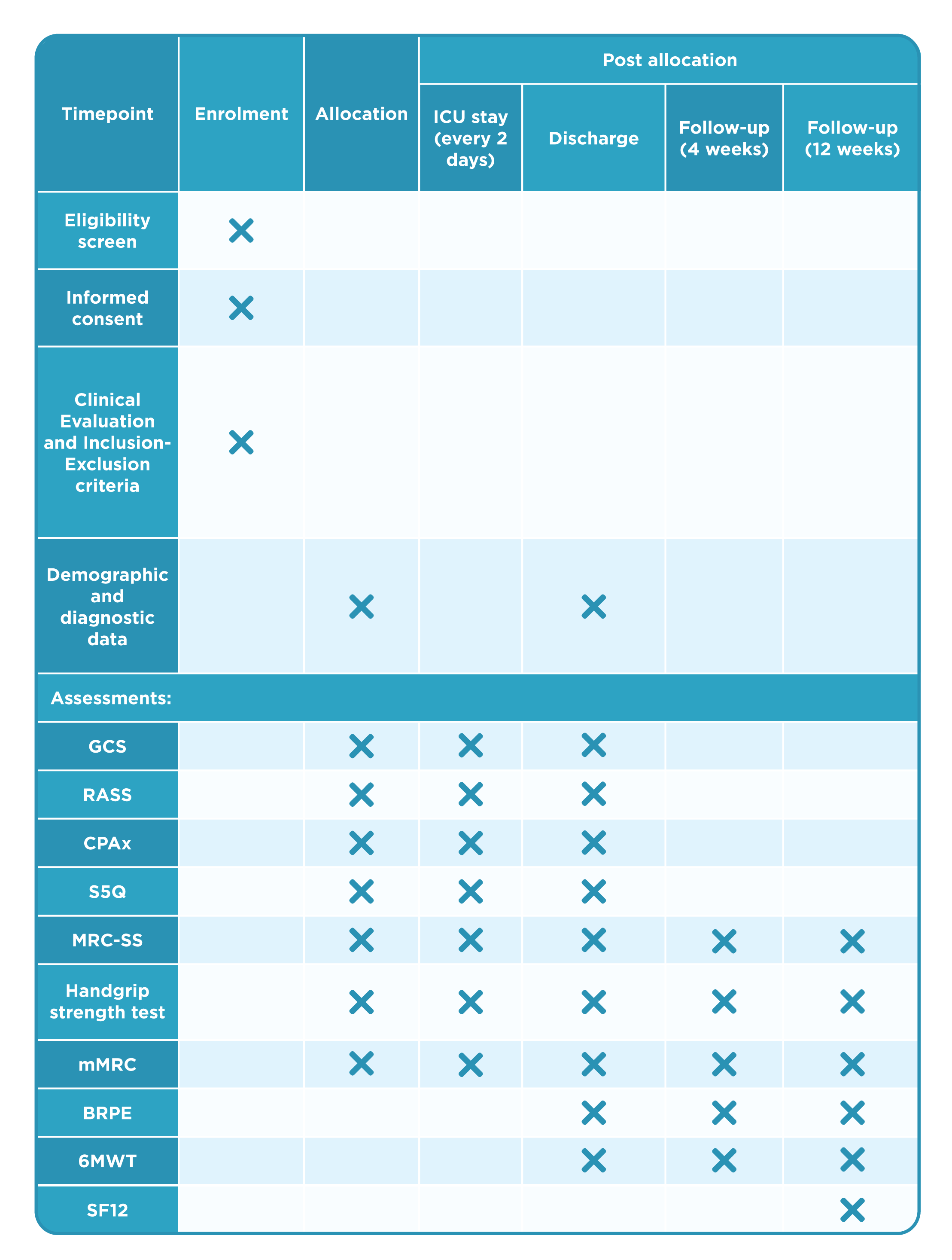


Table 1 – Schedule for enrolment, data collection, intervention and outcome measures. GCS - Glasgow Coma Scale; RASS - Richmond Agitation Sedation Scale; CPAx - Chelsea Critical Care Physical Assessment; S5Q - 5 standardized questions for cooperation; MRC – SS - Medical Research Council sum score; mMRC - Medical Research Council dyspnea scale; BRPE - Borg Rating of Perceived Exertion; 6MWT - 6-Minute Walk Test; SF-12- 12-Item Short Form Survey.

**Statistical Analysis**

The statistical analysis will be carried out through the SSPS®, version 25.0 (SSPS Inc., Chicago, IL, USA). Continuous variables will be expressed as mean and standard deviation or standard error, or as median and interquartile range. Categorical variables will be expressed as absolute and relative frequencies.

The Kolmogorov-Smirnov test will be used to evaluate the normal distribution of the continuous variables. We will analyze the hypothesis contrast inference by Student’s t test for independent variables with parametric distributions and Mann-Whitney’s U for non-parametric distributions. The confidence level used will be 95% (0,05), and the power of the study will be 90% (0,1). If the rate of loss to follow-up were observed greater than 15%, we will perform an intention-to-treat analysis. A sub-analysis will be carried out, depending on the age of the patients, sex, and clinical status.

**Discussion**

As of 31^st^ January 2021, Portugal had 6869 hospitalized patients due to COVID-19, 865 of them in ICU, with increasing tendency (37). This number of ICU patients occupies 84% of the total 1021 ICU vaccancies available in Portugal for all kinds of phatologies. This situation represents a huge burden on the national health system and also raises public health concerns, as care in the ICU is usually intense due to the serious medical conditions themselves, such as the use of life-sustaining equipment, sedatives, pain and other medications that have mind-altering effects, and this exposure to stressors impacts several aspects of the patient (14). The prevention and reduction of PICS is a health problem with growing relevance in this context.

This article presents a detailed description of a randomized controlled trial designed to analyze the results of the implementation of a respiratory and functional rehabilitation program, applied in patients affected by COVID-19 hospitalized in ICU. This trial has the strength of having a pragmatic design of early initiation of a rehabilitation protocol that allows the prospective inclusion of patients, and also the follow-up of participants up to 12 weeks after discharge. The implementation of the protocol, if it proves superior to standard care, in the daily routine of the ICU will be simple, and it could constitute the foundation for the development of new treatment guidelines. Limitations of this study are the intervention being performed only in one center an one-center and the impossibility of blinding the ICU staff, nonetheless the assessors responsible for randomization and outcome measures will be completely blinded to group allocation. The treating professionals have been given formation about the possible adverse events that can happen during the intervention and the importance of reporting them.

We expect the intervention group to achieve better functional and respiratory outcomes, less days of hospitalization and an improvement in health related quality of life, comparing to the standard of care group. The results will be communicated to the Portuguese health authorities internally and to other relevant groups through publications in medical journals.

**Trial status**

This is the second and definitive protocol version. Participants will be recruited between March 8, 2021, and June 8, 2021. Study completion is expected to be October 2021. The study protocol has been submitted before the end of the recruitment and before the last patient.

**Abbreviations**

SARS-CoV - severe acute respiratory syndrome-related coronavirus

SARS-CoV-2 - severe acute respiratory syndrome coronavirus 2

WHO - World Health Organization

COVID – 19 - coronavirus disease 2019

ARDS - acute respiratory distress syndrome

DIC - disseminated intravascular coagulopathy

ICU - intensive care unit

PICS - post-intensive care syndrome

ICU – AW - Intensive care unit-acquired weakness

GCS - Glasgow Coma Scale

RASS - Richmond Agitation Sedation Scale

CPAx - Chelsea Critical Care Physical Assessment

S5Q - 5 standardized questions for cooperation

MRC - Medical Research Council

MRC – SS - Medical Research Council sum score

mMRC - Medical Research Council dyspnea scale

BRPE - Borg Rating of Perceived Exertion

6MWT - 6-Minute Walk Test

HRQoL - The health related quality of life

SF-12- 12-Item Short Form Survey

CMG - Case Mix Groups

**Acknowledgements**

Not applicable.

**Authors’ contributions**

All the procedures for recruiting, intervening, and evaluating the participants are carried out by personnel attached to this group. ACC is the principal investigator and has contributed to the concept and study design, provided clinical expertise and contributed to the manuscript development. JM, PC, PCA and CAB contributed to the protocol development and provided clinical expertise. BG contributed to the protocol development, provided clinical expertise and he is responsible for designing the statistical procedures. All authors read and approved the final manuscript.

**Funding**

There are no sources of funding.

**Availability of data and materials**

The datasets analyzed during the current study are available from the corresponding author on reasonable request. The data will be available after the main publication of them; for other circumstances, they should consult the corresponding author. Any data required to support the protocol can be supplied on request.

**Ethics approval and consent to participate**

This study complies with the Helsinki guidelines for human research, and it has been approved by the Commission of Ethics for Health of Centro Hospitalar Entre Douro e Vouga, reference CA-081/2021-0t_MP/CC. All study participants signed an informed consent approved by the ethics committee. The identification of each individual will remain concealed based on the ethical principles of confidentiality and privacy. Any reason for compensation will be covered by professional liability insurance. Informed consent is available in the portuguese language from the corresponding author on request. There is no anticipated harm and compensation for trial participation.

**Consent for publication**

Not applicable.

**Competing interests**

The authors declare that they have no competing interests.

**References**

1. Zhu N, Zhang D, Wang W, Li X, Yang B, Song J, et al. A Novel Coronavirus from Patients with Pneumonia in China, 2019. N Engl J Med. 2020;382(8):727-33.

2. Kaye AD, Cornett EM, Brondeel KC, Lerner ZI, Knight HE, Erwin A, et al. Biology of COVID-19 and related viruses: Epidemiology, signs, symptoms, diagnosis, and treatment. Best Pract Res Clin Anaesthesiol. 2020.

3. Wang H, Li X, Li T, Zhang S, Wang L, Wu X, et al. The genetic sequence, origin, and diagnosis of SARS-CoV-2. Eur J Clin Microbiol Infect Dis. 2020;39(9):1629-35.

4. World Health Organization Clinical management of COVID-19: interim guidance, 27 May 2020. Geneva: World Health Organization; 2020. Contract No.: WHO/2019 nCoV/clinical/2020.5.

5. World Health Organization. WHO Director-General's opening remarks at the media briefing on COVID-19 - 11 March 2020 Available from: https://www.who.int/director-general/speeches/detail/who-director-general-s-opening-remarks-at-the-media-briefing-on-covid-19---11-march-2020.

6. Sanche S, Lin YT, Xu C, Romero-Severson E, Hengartner N, Ke R. High Contagiousness and Rapid Spread of Severe Acute Respiratory Syndrome Coronavirus 2. Emerging Infectious Disease journal. 2020;26(7):1470.

7. World Health Organization. Weekly epidemiological update - 27 January 2021 Available from: https://www.who.int/publications/m/item/weekly-epidemiological-update---27-january-2021.

8. Manabe T, Akatsu H, Kotani K, Kudo K. Trends in clinical features of novel coronavirus disease (COVID-19): A systematic review and meta-analysis of studies published from December 2019 to February 2020. Respir Investig. 2020;58(5):409-18.

9. Wiersinga WJ, Rhodes A, Cheng AC, Peacock SJ, Prescott HC. Pathophysiology, Transmission, Diagnosis, and Treatment of Coronavirus Disease 2019 (COVID-19): A Review. JAMA. 2020;324(8):782-93.

10. Pascarella G, Strumia A, Piliego C, Bruno F, Del Buono R, Costa F, et al. COVID-19 diagnosis and management: a comprehensive review. J Intern Med. 2020;288(2):192-206.

11. Huang C, Wang Y, Li X, Ren L, Zhao J, Hu Y, et al. Clinical features of patients infected with 2019 novel coronavirus in Wuhan, China. Lancet. 2020;395(10223):497-506.

12. Stam HJ, Stucki G, Bickenbach J. Covid-19 and Post Intensive Care Syndrome: A Call for Action. J Rehabil Med. 2020;52(4):jrm00044.

13. Needham DM, Davidson J, Cohen H, Hopkins RO, Weinert C, Wunsch H, et al. Improving long-term outcomes after discharge from intensive care unit: report from a stakeholders' conference. Crit Care Med. 2012;40(2):502-9.

14. Inoue S, Hatakeyama J, Kondo Y, Hifumi T, Sakuramoto H, Kawasaki T, et al. Post-intensive care syndrome: its pathophysiology, prevention, and future directions. Acute Med Surg. 2019;6(3):233-46.

15. Smith S, Rahman O. Post Intensive Care Syndrome. StatPearls. Treasure Island (FL): StatPearls Publishing;2020.

16. Hopkins RO, Girard TD. Medical and economic implications of cognitive and psychiatric disability of survivorship. Semin Respir Crit Care Med. 2012;33(4):348-56.

17. Torres J, Carvalho D, Molinos E, Vales C, Ferreira A, Dias CC, et al. The impact of the patient post-intensive care syndrome components upon caregiver burden. Med Intensiva. 2017;41(8):454-60.

18. Kress JP, Hall JB. ICU-acquired weakness and recovery from critical illness. N Engl J Med. 2014;370(17):1626-35.

19. Stevens RD, Dowdy DW, Michaels RK, Mendez-Tellez PA, Pronovost PJ, Needham DM. Neuromuscular dysfunction acquired in critical illness: a systematic review. Intensive Care Med. 2007;33(11):1876-91.

20. Fuke R, Hifumi T, Kondo Y, Hatakeyama J, Takei T, Yamakawa K, et al. Early rehabilitation to prevent postintensive care syndrome in patients with critical illness: a systematic review and meta-analysis. BMJ Open. 2018;8(5):e019998.

21. Tipping CJ, Harrold M, Holland A, Romero L, Nisbet T, Hodgson CL. The effects of active mobilisation and rehabilitation in ICU on mortality and function: a systematic review. Intensive Care Med. 2017;43(2):171-83.

22. Connolly B, Salisbury L, O'Neill B, Geneen L, Douiri A, Grocott MP, et al. Exercise rehabilitation following intensive care unit discharge for recovery from critical illness. Cochrane Database Syst Rev. 2015(6):CD008632.

23. Hosey MM, Needham DM. Survivorship after COVID-19 ICU stay. Nat Rev Dis Primers. 2020;6(1):60-.

24. Candan SA, Elibol N, Abdullahi A. Consideration of prevention and management of long-term consequences of post-acute respiratory distress syndrome in patients with COVID-19. Physiother Theory Pract. 2020;36(6):663-8.

25. Begg C, Cho M, Eastwood S, Horton R, Moher D, Olkin I, et al. Improving the quality of reporting of randomized controlled trials. The CONSORT statement. JAMA. 1996;276(8):637-9.

26. Portuguese Society of Physical and Rehabilitation Medicine. Taskforces SPMFR COVID-19 [Available from: http://www.spmfr.org/taskforce-spmfr-covid-19/.

27. Reith FC, Van den Brande R, Synnot A, Gruen R, Maas AI. The reliability of the Glasgow Coma Scale: a systematic review. Intensive Care Med. 2016;42(1):3-15.

28. Sessler CN, Gosnell MS, Grap MJ, Brophy GM, O'Neal PV, Keane KA, et al. The Richmond Agitation-Sedation Scale: validity and reliability in adult intensive care unit patients. Am J Respir Crit Care Med. 2002;166(10):1338-44.

29. Corner EJ, Wood H, Englebretsen C, Thomas A, Grant RL, Nikoletou D, et al. The Chelsea critical care physical assessment tool (CPAx): validation of an innovative new tool to measure physical morbidity in the general adult critical care population; an observational proof-of-concept pilot study. Physiotherapy. 2013;99(1):33-41.

30. Sommers J, Engelbert RHH, Dettling-Ihnenfeldt D, Gosselink R, Spronk PE, Nollet F, et al. Physiotherapy in the intensive care unit: an evidence-based, expert driven, practical statement and rehabilitation recommendations. Clin Rehabil. 2015;29(11):1051-63.

31. Hermans G, Clerckx B, Vanhullebusch T, Segers J, Vanpee G, Robbeets C, et al. Interobserver agreement of Medical Research Council sum-score and handgrip strength in the intensive care unit. Muscle Nerve. 2012;45(1):18-25.

32. Milačić N, Milačić B, Dunjić O, Milojkovic M. Validity of CAT and MMRC - Dyspnea score in evaluatuion of COPD severity. Acta Medica Medianae. 2015;54:66-70.

33. Gonzalez-Gerez JJ, Bernal-Utrera C, Anarte-Lazo E, Garcia-Vidal JA, Botella-Rico JM, Rodriguez-Blanco C. Therapeutic pulmonary telerehabilitation protocol for patients affected by COVID-19, confined to their homes: study protocol for a randomized controlled trial. Trials. 2020;21(1):588.

34. Enright PL. The six-minute walk test. Respir Care. 2003;48(8):783-5.

35. Ware J, Jr., Kosinski M, Keller SD. A 12-Item Short-Form Health Survey: construction of scales and preliminary tests of reliability and validity. Med Care. 1996;34(3):220-33.

36. Pequeno NPF, Cabral NLA, Marchioni DM, Lima S, Lyra CO. Quality of life assessment instruments for adults: a systematic review of population-based studies. Health Qual Life Outcomes. 2020;18(1):208.

37. Direcção Geral da Saúde. COVID19 - Relatório de Situação Available from: https://covid19.min-saude.pt/wp-content/uploads/2021/02/336_DGS_boletim_20210201.pdf.
